# A novel endoscopic plastic stent exchange technique using a loop cutter in malignant afferent loop obstruction: a method applicable to various pancreatobiliary situations

**DOI:** 10.1055/a-2849-8990

**Published:** 2026-04-24

**Authors:** Akinobu Koiwai, Morihisa Hirota, Nana Inomata, Yuki Miyashita, Takehito Itoh, Kennichi Satoh

**Affiliations:** 1 Division of Gastroenterology 34836 Tohoku Medical and Pharmaceutical UniversitySendai CityJapan

Malignant afferent loop obstruction (ALO) after Billroth-II gastrectomy may cause perforation, cholangitis, or pancreatitis if decompression is delayed 1 2 . A plastic stent (PS) is used for palliation, but once the stent is occluded, re-intervention can be challenging when the afferent limb orifice is obscured 1 3 4 . In such situations, blind stent removal risks loss of access.

**Fig. 1:**
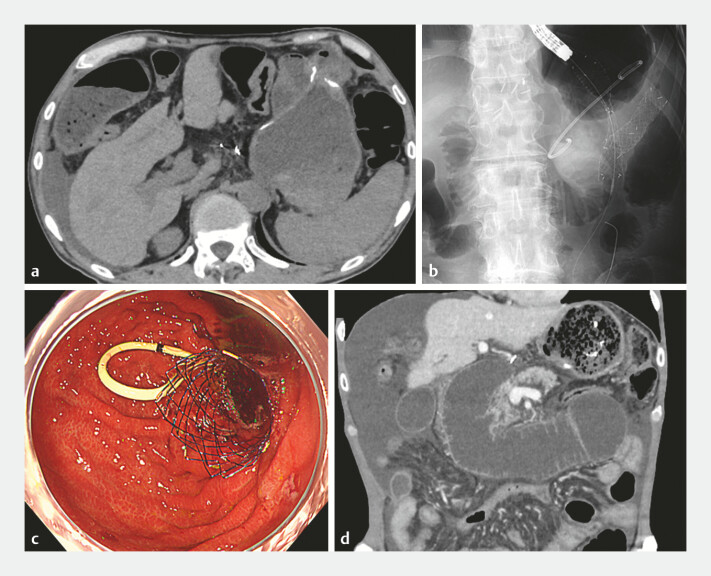
**a** CT showing efferent loop obstruction at the gastrojejunostomy due to peritoneal dissemination. **b** A fluoroscopic image demonstrating the deployment of a SEMS across the efferent limb stricture. **c** An endoscopic view showing the SMES in the efferent limb and the placement of a prophylactic double-pigtail plastic stent in the afferent limb. **d** CT obtained 4 months later showing marked dilation of the afferent loop caused by occlusion of the indwelling plastic stent. CT, computed tomography; SEMS, self-expandable metallic stent.

**Fig. 2:**
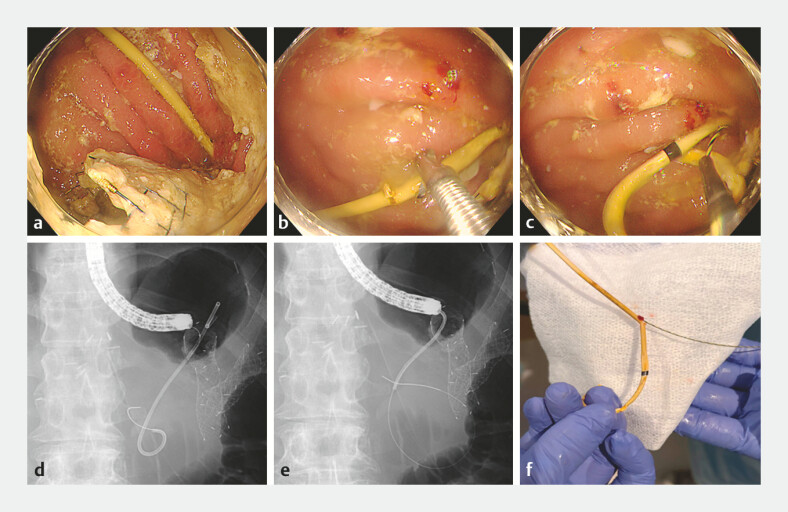
**a–c** Endoscopic images. **a** An endoscopic view showing that the afferent limb orifice was no longer visible because of tumor progression and expansion of the efferent limb metallic stent. **b** A loop cutter was applied to the midportion of the indwelling plastic stent to create a small fenestration. **c** An endoscopic view confirming the passage of a 0.025-inch guidewire through the fenestration into the afferent limb. **d–e** Fluoroscopic images. **d** A fluoroscopic image showing the guidewire advanced through the fenestrated plastic stent into the dilated afferent loop. **e** Fluoroscopic removal of the occluded plastic stent over the indwelling guidewire. **f** Macroscopic appearance of the removed double-pigtail plastic stent, showing the segment that was fenestrated using the loop cutter.

Our patient underwent distal gastrectomy (Billroth-II reconstruction) for gastric cancer. Four years later, peritoneal dissemination caused obstruction. Efferent loop obstruction at the gastrojejunostomy ( Fig. 1 **a** ) was treated with a self-expandable metallic stent (SEMS, HANAROSTENT Naturfit-Duo, BostonScientific, MA, USA; 22 mm × 10 cm), and a prophylactic double-pigtail PS (AdvaniX-J, BostonScientific; 7 Fr × 10 cm) was placed in the afferent loop ( Fig. 1 **b** and **c** ). However, after the placement of the SMES and PS, the patient developed recurrent ALO due to PS occlusion ( Fig. 1 **d** ), and at re-intervention the afferent orifice was no longer visible endoscopically ( Fig. 2 **a** ).

**Fig. 3:**
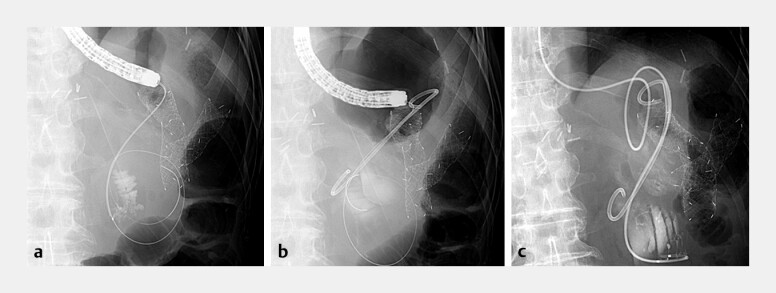
**a** After exchange to a catheter, contrast injection confirms opacification of the afferent limb, demonstrating the correct positioning of the guidewire and catheter. **b** A fluoroscopic image demonstrating the placement of the new double-pigtail stent into the afferent limb. **c** A fluoroscopic image showing the additional placement of a nasobiliary drainage tube alongside the new plastic stent.

Under fluoroscopic guidance, we created a fenestration at the midportion of the indwelling PS using a loop cutter (FS-5Q-1, Olympus, Tokyo, Japan Video image; Fig. 2 b). The PS was not completely transected; leaving a partial hinge maintains alignment and facilitates guidewire direction into the lumen. A 0.025-inch guidewire was then advanced through the fenestration ( Fig. 2 c and d). Once the wire position was confirmed, the occluded PS was removed with forceps ( Fig. 2 e and f), and contrast injection through a catheter confirms correct positioning within the afferent limb ( Fig. 3 a). We placed two guidewires and a new PS and a nasobiliary drainage tube are inserted ( Fig. 3 b and c, Video 1 ).

Loop cutter–assisted fenestration of an indwelling plastic stent allows guidewire passage and safe exchange to a new double-pigtail stent in malignant afferent loop obstruction after Billroth-II gastrectomy.Video 1

This simple technique preserves the existing tract avoiding losing the only safe access route. We consider loop cutter-assisted fenestration as a salvage option not only for malignant ALO but also for PS exchanges in the pancreatobiliary field, such as occluded stents after endoscopic ultrasonography-guided biliary drainage or pancreatic duct drainage.

Endoscopy_UCTN_Code_TTT_1AR_2A
